# Supplementary material for: L-Phenylalanine restriction enhances L-boronophenylalanine uptake and improves boron neutron capture therapy efficacy in tumor cell lines
Source: PLoS One. 2026 Aug 13;21(8):e0355966. doi: 10.1371/journal.pone.0355966 (PMC13472641; doi:10.1371/journal.pone.0355966)
Supplement: Supplementary file 1 [file pone.0355966.s011.pdf]

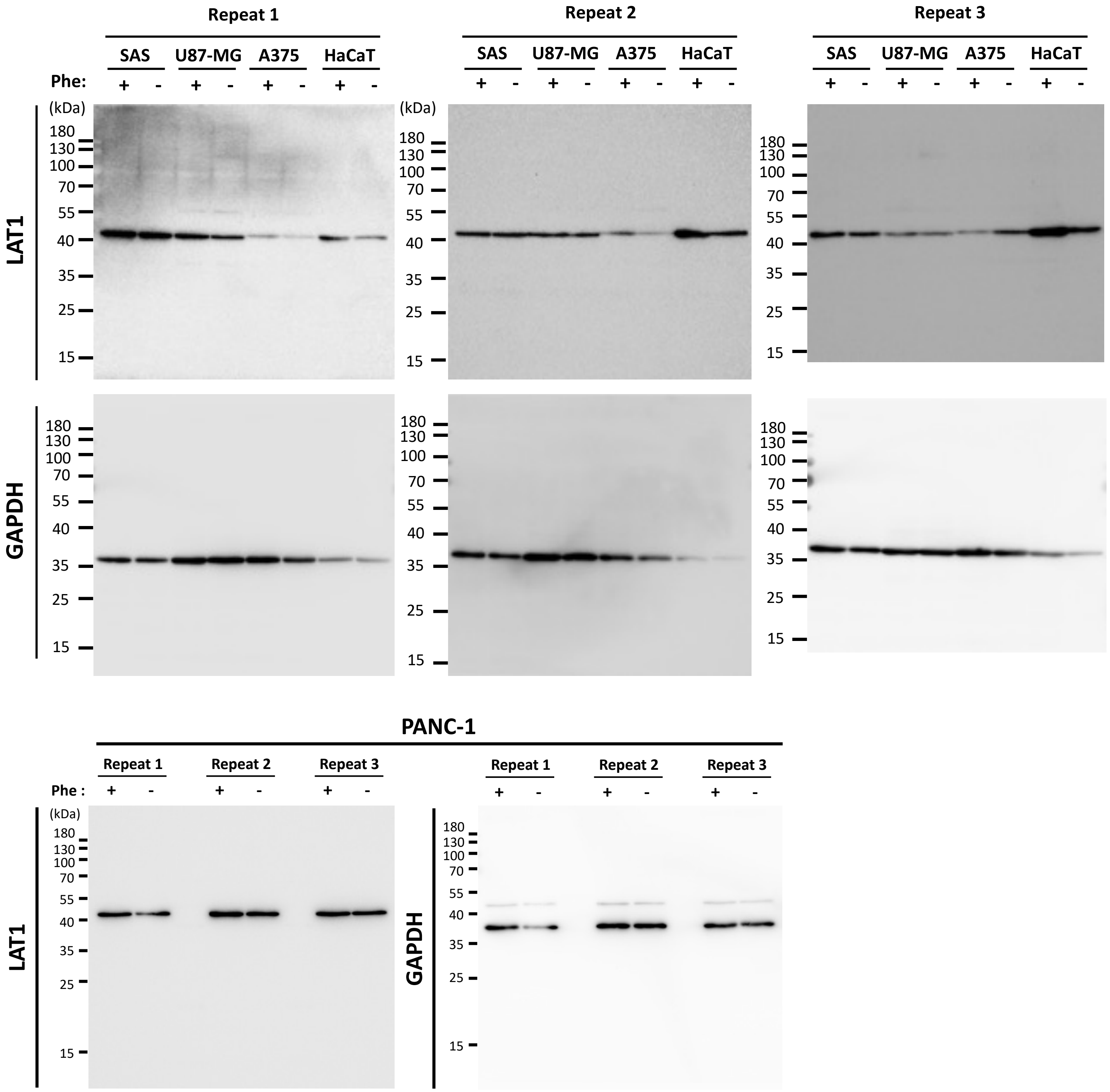

S6 File. Original uncropped and unadjusted western blot images underlying S4C Fig, including replicate experiments.
