# Supplementary material for: L-Phenylalanine restriction enhances L-boronophenylalanine uptake and improves boron neutron capture therapy efficacy in tumor cell lines
Source: PLoS One. 2026 Aug 13;21(8):e0355966. doi: 10.1371/journal.pone.0355966 (PMC13472641; doi:10.1371/journal.pone.0355966)
Supplement: Supplementary file 3 [file pone.0355966.s014.docx]

**Response to Reviewers**

We thank the Editor and the reviewers for their careful evaluation of our manuscript and for their constructive comments. We have revised the manuscript accordingly to improve clarity and reproducibility. All reviewer comments are reproduced below, followed by our responses.

**Reviewer #1**

**Comment:**

The authors have failed significantly to cite the relevant journal publications, along with latest review articles published by Y. Zhu and co-workers. Interestingly, one of the books on Cancer Therapies involving boron and gadolinium is now translated into Traditional Chinese (https://hochi.meepshoper.com/products/5666c220-009b-4e87-bf35-6c608ab8a34c) in 2019 and another on Boron Science has been translated into Turkish language (https://boren.tenmak.gov.tr/tr/duyurular/200-bor-bilim-kitabi-yeni-teknolojiler-ve-uygulamalar.html) in 2022. If the authors type "books on BNCT & GdNCT and Other Medical Applications" in their web browser, they will see many publications (https://www.sciencedirect.com/science/article/pii/S0928493117336846?via%3Dihub; <https://pubs.rsc.org/en/content/articlelanding/2017/nj/c6nj03894h>; https://onlinelibrary.wiley.com/doi/abs/10.1002/zaac.201600181) including many books published in recent years (https://www.ncbi.nlm.nih.gov/pmc/articles/PMC7071021/; https://www.worldscientific.com/worldscibooks/10.1142/q0130#t=aboutBook;https://www.wiley.com/en-us/Boron+Based+Compounds:+Potential+and+Emerging+Applications+in+Medicine-p-9781119275558; https://www.hindawi.com/journals/jchem/si/919356/; <https://www.worldscientific.com/worldscibooks/10.1142/8056>; <https://www.springer.com/us/book/9780306445675>; <https://www.springer.com/us/book/9783642313332>; https://www.crcpress.com/Boron-Science-New-Technologies-and-Applications/Hosmane/p/book/9781439826621; https://hochitw.com/index_down.php?openCatIDfor3=&openCatID=0&CAhs=&firestpageset=1&ISPID=14909&IIBig=71&sele=shopbig_dm_right; https://www.worldscientific.com/doi/10.1142/q0130-vol4). Without the citation of these books, along with relevant journal publications (https://link.springer.com/article/10.1140/epjp/s13360-023-04150-7; <https://www.nature.com/articles/s41598-020-62268-5>), I consider this manuscript is "Incomplete"! Surprisingly, the authors are not at all aware of any of these publications either. However, I leave the final decision of major revision, rejection or accepting this manuscript in its present form to the editor of this journal.

**Response:**

We thank Reviewer #1 for pointing out additional literature related to BNCT research. After reviewing the suggested publications, we have added a reference (Hattori et al., 2023) in the Methods section to further support the analytical approach used in this study (boron quantification by ICP-AES), which is widely applied in BNCT research.

Our study focuses on the experimental evaluation of phenylalanine restriction as a strategy to enhance BPA uptake and BNCT efficacy in specific tumor cell lines. Therefore, we have ensured that key literature directly relevant to BNCT methodology and boron quantification is appropriately cited in the revised manuscript.

In addition, in accordance with the data availability requirements of PLOS ONE, we have revised the Data Availability statement and added supplementary files containing the numerical data underlying the figures as S1–S5 Files. These files are provided as Excel files in the revised submission.

**Reviewer #2**

We thank Reviewer #2 for the careful and constructive evaluation of our manuscript.

**Comment 1:** In the ICP-AES section, an equation is provided to calculate intracellular boron concentration; however, the units on the left- and right-hand sides do not appear to be consistent. In addition, the rationale for the multiplication factor “×4” is unclear. Please revise this section to clearly explain all conversion steps so that the calculation can be readily reproduced.

**Response 1:** In ICP-AES, the boron concentration in the sample solution is calculated in µg/mL. To determine the total boron concentration in the solution, the sample volume must be multiplied. Since the sample volume in this experiment was 4 mL, it was indicated as “×4”. Furthermore, the intracellular boron concentration (mg/1×10⁶ cells) was calculated by dividing the total solution boron concentration by the cell count (×10⁶). As you pointed out, the meaning of “×4” was unclear, so it has been changed to Vsample (mL), with an explanation added. Additionally, “×10⁶” has been added to the cell count to indicate that the units on both sides of the equation are consistent.

**Comment 2:** Some figures appear to involve comparisons across multiple groups or conditions, yet Student’s t-test is indicated in the figure legends. Please clarify the statistical approach in detail and confirm whether this test is appropriate for the experimental design.

**Response 2:** The statistical approach has been clarified in detail in the Methods section. For comparisons between two independent groups (e.g., Phe(+)/Phe(−)), Student’s t-test was used. For experiments involving multiple concentration conditions in S1 Fig, one-way ANOVA followed by appropriate post hoc analysis was applied where necessary. Figure legends have been updated accordingly.

**Comment 3:** Please check the legend for Figure 3. The Results text and the data shown suggest that the 44 commonly affected genes are upregulated, whereas the legend refers to them as “downregulated.” Please correct this inconsistency.

**Response 3:** The inconsistency has been corrected. The 44 commonly affected genes are correctly described as “upregulated”.

**Comment 4:** The Methods section states that thermal neutron fluence and gamma-ray dose were measured using gold foils and TLDs, respectively. However, the actual measured/calculated values are not explicitly reported. Please provide the neutron fluence (and accompanying gamma dose) used in the cell irradiation experiments to ensure reproducibility and to clarify how these values relate to the survival curves shown in Figure 2.

**Response 4:** The measured thermal neutron fluence and accompanying gamma-ray dose used in the irradiation experiments have now been explicitly reported in Table 1 of the revised manuscript. These values correspond to the irradiation conditions used for the colony formation assays shown in Figure 2. The underlying numerical data are provided in S1 File to further improve transparency and reproducibility.

We believe that these revisions have addressed the reviewers’ comments and improved the clarity and reproducibility of the manuscript.

We appreciate your time and consideration.

Sincerely,

Rie Saba, Ph.D.
(on behalf of all authors)
