# Supplementary material for: L-Phenylalanine restriction enhances L-boronophenylalanine uptake and improves boron neutron capture therapy efficacy in tumor cell lines
Source: PLoS One. 2026 Aug 13;21(8):e0355966. doi: 10.1371/journal.pone.0355966 (PMC13472641; doi:10.1371/journal.pone.0355966)
Supplement: Supplementary file 4 [file pone.0355966.s015.docx]

**Response to reviewers for 2^nd^ revision**

We thank the Editor and Reviewer #3 for the careful evaluation of our manuscript and for the constructive comments. We have revised the manuscript accordingly to improve clarity and reproducibility. All reviewer comments are reproduced below, followed by our responses.

**Reviewer #3**

We appreciate Reviewer #3’s careful and constructive comments.

**Comment 1:** The mechanistic conclusion regarding LAT1/4F2HC-mediated exchange transport remains speculative. Although intracellular amino acid alterations were observed, no direct functional validation (e.g., LAT1 inhibition, knockdown, or transport competition assays) was performed. The authors should moderate mechanistic statements throughout the manuscript.

**Response 1:** We agree that our data do not directly demonstrate LAT1/4F2HC-mediated transport. We therefore moderated mechanistic statements throughout the manuscript and clearly state that this mechanism represents a working model based on the observed changes in intracellular amino acid levels and the known transport properties of LAT1/4F2HC (page 15, lines 300–302; page 16, lines 312–315).

**Comment 2:** The clinical relevance of “Phe restriction” should be discussed more carefully. The experimental condition corresponds to complete Phe deprivation in vitro, which may not accurately reflect clinically achievable dietary restriction in patients. In addition, throughout the manuscript, the term “Phe diet” is repeatedly used in the context of cell culture experiments. Since “diet” is generally used for animal or human studies, terms such as “Phe restriction,” “Phe deprivation,” or “Phe-free condition” would be more appropriate.

**Response 2:** We thank the reviewer for this helpful suggestion. Throughout the manuscript, we replaced the term **"Phe diet"** with **"Phe restriction," "Phe deprivation," or "Phe-free condition,"** as appropriate. **We also revised the Discussion to more carefully discuss the potential clinical application of this strategy.**

**Comment 3:** Since the proposed significance of this strategy is improvement of tumor selectivity in BNCT, the absence of in vivo validation should be more explicitly acknowledged as a major limitation.

**Response 3:** We appreciate the reviewer's important comment. We have expanded the Discussion to explicitly acknowledge the lack of in vivo validation as a major limitation of the present study. We now state that whether short-term phenylalanine restriction enhances tumor-selective BPA accumulation and improves BNCT efficacy without increasing BPA uptake in normal tissues remains to be determined through future in vivo studies (page 17, lines 341–346).

**Comment 4:** The heterogeneous responses among tumor cell lines are interesting but insufficiently discussed. Additional interpretation regarding why A375 cells failed to increase BPA uptake under Phe restriction would strengthen the manuscript.

**Response 4:** We thank the reviewer for this insightful comment. We agree that the heterogeneous response observed in A375 cells warrants further investigation. Because the present study was designed to evaluate the effects of phenylalanine restriction on BPA uptake rather than to identify the determinants of cell line-specific responsiveness, we have expanded the Discussion to acknowledge that additional regulatory mechanisms are likely involved and that elucidating these mechanisms will be an important focus of future studies (page 16, lines 312–318).

**Minor Comments (MCs)**
**MC1:** The title is somewhat long and grammatically awkward because “amplifies” and “induces” are used in parallel. In addition, the phrase “activating transcription factor 4 stress response” sounds unnatural. Because ATF4 is not the “stress” itself, but rather a transcription factor that regulates the stress response.

**Response MC1:** We revised the title to “L-Phenylalanine restriction enhances L-boronophenylalanine uptake and improves boron neutron capture therapy efficacy in tumor cell lines” (page 1, lines 1–2).

**MC2:** In the Abstract, the phrase “on modulating BPA uptake and associated cellular responses” is awkward. Please revise this expression to improve grammatical flow.

**Response MC2:** We revised the text “on modulating BPA uptake and associated cellular responses” to “affects BPA uptake and related cellular responses”.

**MC3:** In the Introduction, the sentence beginning with “The active uptake of amino acids via the high-level expression...” is difficult to follow structurally. Please consider simplifying and restructuring this sentence for clarity.

**Response MC3:** We revised the sentence as follows: “The high expression of the L-type amino-acid transporter 1 (LAT1)/4F2 heavy chain (4F2HC) complex, the primary transporter for BPA uptake, drives active amino‑acid import and supports tumor cell proliferation.” (page 3, lines 55–58).

**MC4:** In the Methods section, the phrase “filtered using a syringe filter” is somewhat unnatural in scientific writing. Please consider revising to “filtered through a syringe filter.”

**Response MC4:** We thank the reviewer for this suggestion and revised the text accordingly.

**MC5:** In the Results section, the phrase “responsiveness to a Phe diet” is not appropriate for an in vitro cell culture study.

**Response MC5:** We revised the phrase to “response to Phe restriction”.

**MC6:** In the Results section, the sentence “The Phe(–)_100 ppm group showed greater reduction across all cell lines” is ambiguous because it does not specify what was reduced. Please clarify that this refers to cell survival.

**Response MC6:** We revised the sentence as follows: “In the Phe(–)_BPA 100 ppm group, all cell lines showed greater reductions in survival, most notably SAS, which correlated with increased ¹⁰B accumulation.” (page 12, lines 234–236).

**MC7:** In the Discussion, the sentence beginning with “Our working model suggests...” is overly long and difficult to read. Please consider simplifying this sentence for better readability.

**Response MC7:** We revised the text as follows: “Our working model suggests that BPA uptake under Phe restriction is regulated by the balance between intracellular Phe and other amino acids that serve as exchange substrates for the LAT1/4F2HC complex.” (page 16, line 324–326).

**MC8:** In the Discussion, the sentence “The intracellular Phe levels decreased, which enhanced the uptake of BPA” implies direct causality that has not been fully demonstrated experimentally.

**Response MC8:** We revised the text accordingly, “In SAS cells, increased exchangeable substrates and significantly reduced intracellular Phe were associated with enhanced BPA uptake.” (page 16, line 330–331).

**MC9:** The phrase “may serve as a clinical application” is not natural English usage in this context because the term “clinical application” is not usually used to describe a therapy.

**Response MC9:** We revised the sentence as follows: “The established clinical use of phenylalanine restriction in phenylketonuria supports the feasibility of this approach.” (page 18, line 374–375).

**MC10:** The statement “Short-term Phe restriction has minimal side effects” appears overly strong because no in vivo or clinical safety data are presented in this study. Please revise this claim.

**Response MC10:** We revised the sentence as follows: “These findings suggest that short-term Phe restriction may represent a feasible pretreatment strategy for BNCT. Further validation of these in vitro findings in animal models is needed to establish its safety, feasibility, and tumor selectivity.” (page 18–19, line 375–377).

**MC11:** In the Figure 2 legend, the phrase “enhances the efficacy of neutron irradiation” is somewhat imprecise for a BNCT study.

**Response MC11:** We revised the Figure 2 legend to “Phenylalanine deprivation enhances the cytotoxic effect of BNCT.”
