# Supplementary material for: L-Phenylalanine restriction enhances L-boronophenylalanine uptake and improves boron neutron capture therapy efficacy in tumor cell lines
Source: PLoS One. 2026 Aug 13;21(8):e0355966. doi: 10.1371/journal.pone.0355966 (PMC13472641; doi:10.1371/journal.pone.0355966)
Supplement: Supplementary file 5 [file pone.0355966.s016.docx]

**Response to reviewers for 3^rd^ revision**

We thank the reviewer #3 for this helpful comment. We agree that the original statement was too definitive in the absence of direct functional validation of LAT1-mediated BPA transport. We have therefore revised the sentence in the Abstract as follows: “The LAT1/4F2HC complex was suggested to play a predominant role in BPA transport.”
