# Supplementary material for: L-Phenylalanine restriction enhances L-boronophenylalanine uptake and improves boron neutron capture therapy efficacy in tumor cell lines
Source: PLoS One. 2026 Aug 13;21(8):e0355966. doi: 10.1371/journal.pone.0355966 (PMC13472641; doi:10.1371/journal.pone.0355966)
Supplement: Peer review history

# Peer review: Decision Letter 0

**DOI:** 10.1371/journal.pone.0355966.r001

**Authors:** Abbas Farmany

29 Jan 2026

Dear Dr. Saba,

Thank you for submitting your manuscript to PLOS ONE. After careful consideration, we feel that it has merit but does not fully meet PLOS ONE’s publication criteria as it currently stands. Therefore, we invite you to submit a revised version of the manuscript that addresses the points raised during the review process.

Please submit your revised manuscript by Mar 15 2026 11:59PM. If you will need more time than this to complete your revisions, please reply to this message or contact the journal office at plosone@plos.org. When you're ready to submit your revision, log on to https://www.editorialmanager.com/pone/ and select the 'Submissions Needing Revision' folder to locate your manuscript file.

- A letter that responds to each point raised by the academic editor and reviewer(s). You should upload this letter as a separate file labeled 'Response to Reviewers'.
- A marked-up copy of your manuscript that highlights changes made to the original version. You should upload this as a separate file labeled 'Revised Manuscript with Track Changes'.
- An unmarked version of your revised paper without tracked changes. You should upload this as a separate file labeled 'Manuscript'.

If you would like to make changes to your financial disclosure, please include your updated statement in your cover letter. Guidelines for resubmitting your figure files are available below the reviewer comments at the end of this letter.

If applicable, we recommend that you deposit your laboratory protocols in protocols.io to enhance the reproducibility of your results. Protocols.io assigns your protocol its own identifier (DOI) so that it can be cited independently in the future. For instructions see: https://journals.plos.org/plosone/s/submission-guidelines#loc-laboratory-protocols. Additionally, PLOS ONE offers an option for publishing peer-reviewed Lab Protocol articles, which describe protocols hosted on protocols.io. Read more information on sharing protocols at https://plos.org/protocols?utm_medium=editorial-email&utm_source=authorletters&utm_campaign=protocols.

We look forward to receiving your revised manuscript.

Kind regards,

Abbas Farmany

Academic Editor

PLOS One

Journal Requirements:

When submitting your revision, we need you to address these additional requirements.

1. Please ensure that your manuscript meets PLOS ONE's style requirements, including those for file naming. The PLOS ONE style templates can be found at

https://journals.plos.org/plosone/s/file?id=wjVg/PLOSOne_formatting_sample_main_body.pdf and

https://journals.plos.org/plosone/s/file?id=ba62/PLOSOne_formatting_sample_title_authors_affiliations.pdf

2. Thank you for stating the following financial disclosure:

“Y.T.

23K14924

Japan Society for the Promotion of Science Grant-in Aid for Scientific Research

https://www.jsps.go.jp/english/e-grants/

H.Y.

22K07751, 25K02607

Japan Society for the Promotion of Science Grant-in Aid for Scientific Research

https://www.jsps.go.jp/english/e-grants/”

Please state what role the funders took in the study.  If the funders had no role, please state: "The funders had no role in study design, data collection and analysis, decision to publish, or preparation of the manuscript."

If this statement is not correct you must amend it as needed.

Please include this amended Role of Funder statement in your cover letter; we will change the online submission form on your behalf.

3. Please include captions for your Supporting Information files at the end of your manuscript, and update any in-text citations to match accordingly. Please see our Supporting Information guidelines for more information: http://journals.plos.org/plosone/s/supporting-information.

4. PLOS ONE now requires that authors provide the original uncropped and unadjusted images underlying all blot or gel results reported in a submission’s figures or Supporting Information files. This policy and the journal’s other requirements for blot/gel reporting and figure preparation are described in detail at https://journals.plos.org/plosone/s/figures#loc-blot-and-gel-reporting-requirements and https://journals.plos.org/plosone/s/figures#loc-preparing-figures-from-image-files. When you submit your revised manuscript, please ensure that your figures adhere fully to these guidelines and provide the original underlying images for all blot or gel data reported in your submission. See the following link for instructions on providing the original image data: https://journals.plos.org/plosone/s/figures#loc-original-images-for-blots-and-gels.

In your cover letter, please note whether your blot/gel image data are in Supporting Information or posted at a public data repository, provide the repository URL if relevant, and provide specific details as to which raw blot/gel images, if any, are not available. Email us at plosone@plos.org if you have any questions.

5. If the reviewer comments include a recommendation to cite specific previously published works, please review and evaluate these publications to determine whether they are relevant and should be cited. There is no requirement to cite these works unless the editor has indicated otherwise.

[Note: HTML markup is below. Please do not edit.]

Reviewers' comments:

Reviewer's Responses to Questions

**Comments to the Author**

1. Is the manuscript technically sound, and do the data support the conclusions?

Reviewer #1: Partly

Reviewer #2: Yes

**********

2. Has the statistical analysis been performed appropriately and rigorously?

Reviewer #1: I Don't Know

Reviewer #2: N/A

**********

3. Have the authors made all data underlying the findings in their manuscript fully available?

The PLOS Data policy

Reviewer #1: No

Reviewer #2: Yes

**********

4. Is the manuscript presented in an intelligible fashion and written in standard English?

Reviewer #1: No

Reviewer #2: Yes

**********

Reviewer #1: The authors have failed significantly to cite the relevant journal publications, along with latest review articles published by Y. Zhu and co-workers. Interestingly, one of the books on Cancer Therapies involving boron and gadolinium is now translated into Traditional Chinese (https://hochi.meepshoper.com/products/5666c220-009b-4e87-bf35-6c608ab8a34c) in 2019 and another on Boron Science has been translated into Turkish language (https://boren.tenmak.gov.tr/tr/duyurular/200-bor-bilim-kitabi-yeni-teknolojiler-ve-uygulamalar.html) in 2022. If the authors type "books on BNCT & GdNCT and Other Medical Applications" in their web browser, they will see many publications (https://www.sciencedirect.com/science/article/pii/S0928493117336846?via%3Dihub; https://pubs.rsc.org/en/content/articlelanding/2017/nj/c6nj03894h; https://onlinelibrary.wiley.com/doi/abs/10.1002/zaac.201600181) including many books published in recent years (https://www.ncbi.nlm.nih.gov/pmc/articles/PMC7071021/; https://www.worldscientific.com/worldscibooks/10.1142/q0130#t=aboutBook;https://www.wiley.com/en-us/Boron+Based+Compounds:+Potential+and+Emerging+Applications+in+Medicine-p-9781119275558; https://www.hindawi.com/journals/jchem/si/919356/; https://www.worldscientific.com/worldscibooks/10.1142/8056; https://www.springer.com/us/book/9780306445675; https://www.springer.com/us/book/9783642313332; https://www.crcpress.com/Boron-Science-New-Technologies-and-Applications/Hosmane/p/book/9781439826621; https://hochitw.com/index_down.php?openCatIDfor3=&openCatID=0&CAhs=&firestpageset=1&ISPID=14909&IIBig=71&sele=shopbig_dm_right; https://www.worldscientific.com/doi/10.1142/q0130-vol4). Without the citation of these books, along with relevant journal publications (https://link.springer.com/article/10.1140/epjp/s13360-023-04150-7; https://www.nature.com/articles/s41598-020-62268-5), I consider this manuscript is "Incomplete"! Surprisingly, the authors are not at all aware of any of these publications either. However, I leave the final decision of major revision, rejection or accepting this manuscript in its present form to the editor of this journal.

Reviewer #2: This manuscript presents a carefully conducted in vitro study examining the effect of short-term Phe restriction on BPA uptake and BNCT efficacy. The experimental work is generally sound, and the conclusions are largely supported by the data. My specific comments are as follows:

1. In the ICP-AES section, an equation is provided to calculate intracellular boron concentration; however, the units on the left- and right-hand sides do not appear to be consistent. In addition, the rationale for the multiplication factor “×4” is unclear. Please revise this section to clearly explain all conversion steps so that the calculation can be readily reproduced.

2. Some figures appear to involve comparisons across multiple groups or conditions, yet Student’s t-test is indicated in the figure legends. Please clarify the statistical approach in detail and confirm whether this test is appropriate for the experimental design.

3. Please check the legend for Figure 3. The Results text and the data shown suggest that the 44 commonly affected genes are upregulated, whereas the legend refers to them as “downregulated.” Please correct this inconsistency.

4. The Methods section states that thermal neutron fluence and gamma-ray dose were measured using gold foils and TLDs, respectively. However, the actual measured/calculated values are not explicitly reported. Please provide the neutron fluence (and accompanying gamma dose) used in the cell irradiation experiments to ensure reproducibility and to clarify how these values relate to the survival curves shown in Figure 2.

**********

what does this mean?). If published, this will include your full peer review and any attached files.

If you choose “no”, your identity will remain anonymous but your review may still be made public.

**Do you want your identity to be public for this peer review?**  For information about this choice, including consent withdrawal, please see our Privacy Policy

Reviewer #1: No

Reviewer #2: No

**********

[NOTE: If reviewer comments were submitted as an attachment file, they will be attached to this email and accessible via the submission site. Please log into your account, locate the manuscript record, and check for the action link "View Attachments". If this link does not appear, there are no attachment files.]

To ensure your figures meet our technical requirements, please review our figure guidelines: https://journals.plos.org/plosone/s/figures

You may also use PLOS’s free figure tool, NAAS, to help you prepare publication quality figures: https://journals.plos.org/plosone/s/figures#loc-tools-for-figure-preparation.

NAAS will assess whether your figures meet our technical requirements by comparing each figure against our figure specifications.

# Author comment: Author response to Decision Letter 1

**DOI:** 10.1371/journal.pone.0355966.r002

16 Mar 2026

We thank the reviewers and the editor for their constructive comments. Detailed responses to all comments are provided in the attached “Response to Reviewers” document, and the manuscript has been revised accordingly.

Attachment Submitted filename: Response to Reviewers.docx

# Peer review: Decision Letter 1

**DOI:** 10.1371/journal.pone.0355966.r003

**Authors:** Abbas Farmany

31 May 2026

Dear Dr. Saba,

Thank you for submitting your manuscript to PLOS ONE. After careful consideration, we feel that it has merit but does not fully meet PLOS ONE’s publication criteria as it currently stands. Therefore, we invite you to submit a revised version of the manuscript that addresses the points raised during the review process.

Please submit your revised manuscript by Jul 15 2026 11:59PM. If you will need more time than this to complete your revisions, please reply to this message or contact the journal office at plosone@plos.org. When you're ready to submit your revision, log on to https://www.editorialmanager.com/pone/ and select the 'Submissions Needing Revision' folder to locate your manuscript file.

- A letter that responds to each point raised by the academic editor and reviewer(s). You should upload this letter as a separate file labeled 'Response to Reviewers'.
- A marked-up copy of your manuscript that highlights changes made to the original version. You should upload this as a separate file labeled 'Revised Manuscript with Track Changes'.
- An unmarked version of your revised paper without tracked changes. You should upload this as a separate file labeled 'Manuscript'.

If you would like to make changes to your financial disclosure, please include your updated statement in your cover letter. Guidelines for resubmitting your figure files are available below the reviewer comments at the end of this letter.

If applicable, we recommend that you deposit your laboratory protocols in protocols.io to enhance the reproducibility of your results. Protocols.io assigns your protocol its own identifier (DOI) so that it can be cited independently in the future. For instructions see: https://journals.plos.org/plosone/s/submission-guidelines#loc-laboratory-protocols. Additionally, PLOS ONE offers an option for publishing peer-reviewed Lab Protocol articles, which describe protocols hosted on protocols.io. Read more information on sharing protocols at https://plos.org/protocols?utm_medium=editorial-email&utm_source=authorletters&utm_campaign=protocols.

As the corresponding author, your ORCID iD is verified in the submission system and will appear in the published article. PLOS supports the use of ORCID, and we encourage all coauthors to register for an ORCID iD and use it as well. Please encourage your coauthors to verify their ORCID iD within the submission system before final acceptance, as unverified ORCID iDs will not appear in the published article. *Only*  the individual author can complete the verification step; PLOS staff *cannot*  verify ORCID iDs on behalf of authors.

We look forward to receiving your revised manuscript.

Kind regards,

Abbas Farmany

Academic Editor

PLOS One

**Journal Requirements:**

If the reviewer comments include a recommendation to cite specific previously published works, please review and evaluate these publications to determine whether they are relevant and should be cited. There is no requirement to cite these works unless the editor has indicated otherwise.

[Note: HTML markup is below. Please do not edit.]

Reviewers' comments:

Reviewer's Responses to Questions

**Comments to the Author**

Reviewer #1: (No Response)

Reviewer #2: All comments have been addressed

Reviewer #3: All comments have been addressed

**********

2. Is the manuscript technically sound, and do the data support the conclusions?

Reviewer #1: Partly

Reviewer #2: Yes

Reviewer #3: Partly

**********

3. Has the statistical analysis been performed appropriately and rigorously?

Reviewer #1: I Don't Know

Reviewer #2: Yes

Reviewer #3: Yes

**********

4. Have the authors made all data underlying the findings in their manuscript fully available?

The PLOS Data policy

Reviewer #1: No

Reviewer #2: Yes

Reviewer #3: Yes

**********

5. Is the manuscript presented in an intelligible fashion and written in standard English?

Reviewer #1: No

Reviewer #2: Yes

Reviewer #3: Yes

**********

**Reviewer #1:**  It is clear that the authors refused to add the recommended citations and, therefore, this revised version does not qualify again for publication in this journal.

**Reviewer #2:**  (No Response)

**Reviewer #3:**  This manuscript investigates whether phenylalanine restriction enhances BPA uptake and BNCT efficacy in tumor cell lines. The study is technically sound and includes ICP-AES, neutron irradiation assays, RNA-seq, and metabolomic analyses. The findings are interesting and may provide a useful experimental basis for metabolic modulation strategies in BNCT. Therefore, I basically recommend the manuscript suitable for publication in PLOS One. However, several points should be clarified before publication:

1) The mechanistic conclusion regarding LAT1/4F2HC-mediated exchange transport remains speculative. Although intracellular amino acid alterations were observed, no direct functional validation (e.g., LAT1 inhibition, knockdown, or transport competition assays) was performed. The authors should moderate mechanistic statements throughout the manuscript.

2) The clinical relevance of “Phe restriction” should be discussed more carefully. The experimental condition corresponds to complete Phe deprivation in vitro, which may not accurately reflect clinically achievable dietary restriction in patients. In addition, throughout the manuscript, the term “Phe diet” is repeatedly used in the context of cell culture experiments. Since “diet” is generally used for animal or human studies, terms such as “Phe restriction,” “Phe deprivation,” or “Phe-free condition” would be more appropriate.

3) Since the proposed significance of this strategy is improvement of tumor selectivity in BNCT, the absence of in vivo validation should be more explicitly acknowledged as a major limitation.

4) The heterogeneous responses among tumor cell lines are interesting but insufficiently discussed. Additional interpretation regarding why A375 cells failed to increase BPA uptake under Phe restriction would strengthen the manuscript.

Minor Comments

1. The title is somewhat long and grammatically awkward because “amplifies” and “induces” are used in parallel. In addition, the phrase “activating transcription factor 4 stress response” sounds unnatural. Because ATF4 is not the “stress” itself, but rather a transcription factor that regulates the stress response.

2. In the Abstract, the phrase “on modulating BPA uptake and associated cellular responses” is awkward. Please revise this expression to improve grammatical flow.

3. In the Introduction, the sentence beginning with “The active uptake of amino acids via the high-level expression...” is difficult to follow structurally. Please consider simplifying and restructuring this sentence for clarity.

4. In the Methods section, the phrase “filtered using a syringe filter” is somewhat unnatural in scientific writing. Please consider revising to “filtered through a syringe filter.”

5. In the Results section, the phrase “responsiveness to a Phe diet” is not appropriate for an in vitro cell culture study.

6. In the Results section, the sentence “The Phe(–)_100 ppm group showed greater reduction across all cell lines” is ambiguous because it does not specify what was reduced. Please clarify that this refers to cell survival.

7. In the Discussion, the sentence beginning with “Our working model suggests...” is overly long and difficult to read. Please consider simplifying this sentence for better readability.

8. In the Discussion, the sentence “The intracellular Phe levels decreased, which enhanced the uptake of BPA” implies direct causality that has not been fully demonstrated experimentally.

9. The phrase “may serve as a clinical application” is not natural English usage in this context because the term “clinical application” is not usually used to describe a therapy.

10. The statement “Short-term Phe restriction has minimal side effects” appears overly strong because no in vivo or clinical safety data are presented in this study. Please revise this claim.

11. In the Figure 2 legend, the phrase “enhances the efficacy of neutron irradiation” is somewhat imprecise for a BNCT study.

**********

what does this mean?). If published, this will include your full peer review and any attached files.

If you choose “no”, your identity will remain anonymous but your review may still be made public.

**Do you want your identity to be public for this peer review?**  For information about this choice, including consent withdrawal, please see our Privacy Policy

Reviewer #1: No

Reviewer #2: No

Reviewer #3: No

**********

[NOTE: If reviewer comments were submitted as an attachment file, they will be attached to this email and accessible via the submission site. Please log into your account, locate the manuscript record, and check for the action link "View Attachments". If this link does not appear, there are no attachment files.]

To ensure your figures meet our technical requirements, please review our figure guidelines: https://journals.plos.org/plosone/s/figures

You may also use PLOS’s free figure tool, NAAS, to help you prepare publication quality figures: https://journals.plos.org/plosone/s/figures#loc-tools-for-figure-preparation.

NAAS will assess whether your figures meet our technical requirements by comparing each figure against our figure specifications.

# Author comment: Author response to Decision Letter 2

**DOI:** 10.1371/journal.pone.0355966.r004

27 Jun 2026

We thank the Academic Editor and Reviewer #3 for their constructive comments. We have carefully revised the manuscript in response to all comments raised during this round of review. A detailed point-by-point response is provided in the uploaded "Response to Reviewers" document, and all corresponding changes have been incorporated into both the marked-up and clean versions of the revised manuscript.

Attachment Submitted filename: Response to reviewers_2R_v2.4.docx

# Peer review: Decision Letter 2

**DOI:** 10.1371/journal.pone.0355966.r005

**Authors:** Abbas Farmany

24 Jul 2026

Dear Dr. Saba,

Thank you for submitting your manuscript to PLOS ONE. After careful consideration, we feel that it has merit but does not fully meet PLOS ONE’s publication criteria as it currently stands. Therefore, we invite you to submit a revised version of the manuscript that addresses the points raised during the review process.

Please submit your revised manuscript by Sep 07 2026 11:59PM. If you will need more time than this to complete your revisions, please reply to this message or contact the journal office at plosone@plos.org. When you're ready to submit your revision, log on to https://www.editorialmanager.com/pone/ and select the 'Submissions Needing Revision' folder to locate your manuscript file.

- A letter that responds to each point raised by the academic editor and reviewer(s). You should upload this letter as a separate file labeled 'Response to Reviewers'.
- A marked-up copy of your manuscript that highlights changes made to the original version. You should upload this as a separate file labeled 'Revised Manuscript with Track Changes'.
- An unmarked version of your revised paper without tracked changes. You should upload this as a separate file labeled 'Manuscript'.

If you would like to make changes to your financial disclosure, please include your updated statement in your cover letter. Guidelines for resubmitting your figure files are available below the reviewer comments at the end of this letter.

If applicable, we recommend that you deposit your laboratory protocols in protocols.io to enhance the reproducibility of your results. Protocols.io assigns your protocol its own identifier (DOI) so that it can be cited independently in the future. For instructions see: https://journals.plos.org/plosone/s/submission-guidelines#loc-laboratory-protocols. Additionally, PLOS ONE offers an option for publishing peer-reviewed Lab Protocol articles, which describe protocols hosted on protocols.io. Read more information on sharing protocols at https://plos.org/protocols?utm_medium=editorial-email&utm_source=authorletters&utm_campaign=protocols.

As the corresponding author, your ORCID iD is verified in the submission system and will appear in the published article. PLOS supports the use of ORCID, and we encourage all coauthors to register for an ORCID iD and use it as well. Please encourage your coauthors to verify their ORCID iD within the submission system before final acceptance, as unverified ORCID iDs will not appear in the published article. *Only*  the individual author can complete the verification step; PLOS staff *cannot*  verify ORCID iDs on behalf of authors.

We look forward to receiving your revised manuscript.

Kind regards,

Abbas Farmany

Academic Editor

PLOS One

Journal Requirements:

1. If the reviewer comments include a recommendation to cite specific previously published works, please review and evaluate these publications to determine whether they are relevant and should be cited. There is no requirement to cite these works unless the editor has indicated otherwise.

2. Please review your reference list to ensure that it is complete and correct. If you have cited papers that have been retracted, please include the rationale for doing so in the manuscript text, or remove these references and replace them with relevant current references. Any changes to the reference list should be mentioned in the rebuttal letter that accompanies your revised manuscript. If you need to cite a retracted article, indicate the article’s retracted status in the References list and also include a citation and full reference for the retraction notice.

[Note: HTML markup is below. Please do not edit.]

Reviewers' comments:

Reviewer's Responses to Questions

**Comments to the Author**

Reviewer #3: All comments have been addressed

**********

2. Is the manuscript technically sound, and do the data support the conclusions?

Reviewer #3: Yes

**********

3. Has the statistical analysis been performed appropriately and rigorously?

Reviewer #3: Yes

**********

4. Have the authors made all data underlying the findings in their manuscript fully available?

The PLOS Data policy

Reviewer #3: Yes

**********

5. Is the manuscript presented in an intelligible fashion and written in standard English?

Reviewer #3: Yes

**********

Reviewer #3: I appreciate the authors' careful and thoughtful responses to my previous comments. The revised manuscript has been substantially improved, and the major concerns raised in the previous review have been adequately addressed. In particular, the authors have moderated the mechanistic interpretation, clarified the terminology regarding phenylalanine restriction, explicitly acknowledged the lack of in vivo validation as a limitation, and expanded the discussion of the heterogeneous responses among different tumor cell lines. I believe the manuscript is now suitable for publication in PLOS ONE. I have only one minor textual comment that should be addressed before acceptance.

In the abstract, the statement that "the LAT1/4F2HC complex was identified as the sole transport pathway for BPA" goes beyond the scope of the presented experimental evidence. As no direct functional validation of LAT1-mediated transport (such as inhibition, knockdown, or transport competition assays) was performed, this conclusion should be expressed more cautiously. Therefore, I recommend that the authors revise this sentence to indicate that the data support a predominant role for the LAT1/4F2HC complex in BPA transport or suggest its involvement, rather than stating that it was identified as the sole transport pathway.

**********

what does this mean?). If published, this will include your full peer review and any attached files.

If you choose “no”, your identity will remain anonymous but your review may still be made public.

**Do you want your identity to be public for this peer review?**  For information about this choice, including consent withdrawal, please see our Privacy Policy

Reviewer #3: No

**********

[NOTE: If reviewer comments were submitted as an attachment file, they will be attached to this email and accessible via the submission site. Please log into your account, locate the manuscript record, and check for the action link "View Attachments". If this link does not appear, there are no attachment files.]

To ensure your figures meet our technical requirements, please review our figure guidelines: https://journals.plos.org/plosone/s/figures

You may also use PLOS’s free figure tool, NAAS, to help you prepare publication quality figures: https://journals.plos.org/plosone/s/figures#loc-tools-for-figure-preparation.

NAAS will assess whether your figures meet our technical requirements by comparing each figure against our figure specifications.

# Author comment: Author response to Decision Letter 3

**DOI:** 10.1371/journal.pone.0355966.r006

27 Jul 2026

We thank the Academic Editor and the reviewers for their careful evaluation of our manuscript and their constructive comments. We have revised the manuscript accordingly and have provided detailed point-by-point responses to all comments in the attached “Response to reviewers_PONE-D-25-65424R2” file. All changes made in the revised manuscript are described in our responses.

Attachment Submitted filename: Response to reviewers_PONE-D-25-65424R2.docx

# Editor report: Decision Letter 3

**DOI:** 10.1371/journal.pone.0355966.r007

**Authors:** Abbas Farmany

28 Jul 2026

L-Phenylalanine restriction enhances L-boronophenylalanine uptake and improves boron neutron capture therapy efficacy in tumor cell lines

PONE-D-25-65424R3

Dear Dr. Saba,

We’re pleased to inform you that your manuscript has been judged scientifically suitable for publication and will be formally accepted for publication once it meets all outstanding technical requirements.

Within one week, you’ll receive an e-mail detailing the required amendments. When these have been addressed, you’ll receive a formal acceptance letter and your manuscript will be scheduled for publication.

An invoice will be generated when your article is formally accepted. Please note, if your institution has a publishing partnership with PLOS and your article meets the relevant criteria, all or part of your publication costs will be covered. Please make sure your user information is up-to-date by logging into Editorial Manager at Editorial Manager® and clicking the ‘Update My Information' link at the top of the page. For questions related to billing, please contact billing support.

If your institution or institutions have a press office, please notify them about your upcoming paper to help maximize its impact. If they’ll be preparing press materials, please inform our press team as soon as possible -- no later than 48 hours after receiving the formal acceptance. Your manuscript will remain under strict press embargo until 2 pm Eastern Time on the date of publication. For more information, please contact onepress@plos.org.

Kind regards,

Abbas Farmany

Academic Editor

PLOS One

Additional Editor Comments (optional):

Reviewers' comments:

# Editor report: Acceptance letter

**DOI:** 10.1371/journal.pone.0355966.r008

**Authors:** Abbas Farmany

PONE-D-25-65424R3

PLOS One

Dear Dr. Saba,

I'm pleased to inform you that your manuscript has been deemed suitable for publication in PLOS One. Congratulations! Your manuscript is now being handed over to our production team.

At this stage, our production department will prepare your paper for publication. This includes ensuring the following:

* All references, tables, and figures are properly cited

* All relevant supporting information is included in the manuscript submission,

* There are no issues that prevent the paper from being properly typeset

You will receive further instructions from the production team, including instructions on how to review your proof when it is ready. Please keep in mind that we are working through a large volume of accepted articles, so please give us a few days to review your paper and let you know the next and final steps.

Lastly, if your institution or institutions have a press office, please let them know about your upcoming paper now to help maximize its impact. If they'll be preparing press materials, please inform our press team within the next 48 hours. Your manuscript will remain under strict press embargo until 2 pm Eastern Time on the date of publication. For more information, please contact onepress@plos.org.

You will receive an invoice from PLOS for your publication fee after your manuscript has reached the completed accept phase. If you receive an email requesting payment before acceptance or for any other service, this may be a phishing scheme. Learn how to identify phishing emails and protect your accounts at https://explore.plos.org/phishing.

If we can help with anything else, please email us at customercare@plos.org.

Thank you for submitting your work to PLOS ONE and supporting open access.

Kind regards,

PLOS ONE Editorial Office Staff

on behalf of

Dr. Abbas Farmany

Academic Editor

PLOS One
